# Experimental and Numerical Modeling of Aerosol Delivery for Preterm Infants

**DOI:** 10.3390/ijerph15030423

**Published:** 2018-02-28

**Authors:** Iñigo Aramendia, Unai Fernandez-Gamiz, Alberto Lopez-Arraiza, Carmen Rey-Santano, Victoria Mielgo, Francisco Jose Basterretxea, Javier Sancho, Miguel Angel Gomez-Solaetxe

**Affiliations:** 1 Nuclear Engineering and Fluid Mechanics Department, University of the Basque Country UPV/EHU, 01006 Vitoria-Gasteiz, Araba, Spain; unai.fernandez@ehu.eus (U.F.-G.); javier.sancho@ehu.eus (J.S.); 2 Department of Nautical Science and Marine Systems, University of the Basque Country UPV/EHU, 48013 Portugalete, Bizkaia, Spain; alberto.lopeza@ehu.eus (A.L.-A.); miguel.solaetxe@ehu.eus (M.A.G.-S.); 3 Animal Research Unit, BioCruces Health Research Institute, 48903 Barakaldo, Bizkaia, Spain; mariacarmen.reysantano@osakidetza.eus (C.R.-S.); victoriaeugenia.mielgoturuelo@osakidetza.eus (V.M.); 4 Department of Physical Chemistry, University of the Basque Country UPV/EHU, 48940 Leioa, Bizkaia, Spain; franciscojose.basterretxea@ehu.eus

**Keywords:** aerosol, CFD, inhalation catheter, perfluorocarbons, respiratory distress syndrome

## Abstract

Respiratory distress syndrome (RDS) represents one of the major causes of mortality among preterm infants, and the best approach to treat it is an open research issue. The use of perfluorocarbons (PFC) along with non-invasive respiratory support techniques has proven the usefulness of PFC as a complementary substance to achieve a more homogeneous surfactant distribution. The aim of this work was to study the inhaled particles generated by means of an intracorporeal inhalation catheter, evaluating the size and mass distribution of different PFC aerosols. In this article, we discuss different experiments with the PFC perfluorodecalin (PFD) and FC75 with a driving pressure of 4–5 bar, evaluating properties such as the aerodynamic diameter (D_a_), since its value is directly linked to particle deposition in the lung. Furthermore, we develop a numerical model with computational fluid dynamics (CFD) techniques. The computational results showed an accurate prediction of the airflow axial velocity at different downstream positions when compared with the data gathered from the real experiments. The numerical validation of the cumulative mass distribution for PFD particles also confirmed a closer match with the experimental data measured at the optimal distance of 60 mm from the catheter tip. In the case of FC75, the cumulative mass fraction for particles above 10 µm was considerable higher with a driving pressure of 5 bar. These numerical models could be a helpful tool to assist parametric studies of new non-invasive devices for the treatment of RDS in preterm infants.

## 1. Introduction

Complications related to preterm birth are the leading cause of death in children under five years of age. According to data of the World Health Organization (WHO), every year an estimated 15 million babies are born preterm, i.e., before the first 37 weeks of pregnancy. These babies present complications due to the immaturity of their lungs, resulting in a lack of pulmonary surfactant in the respiratory tract and the appearance of the so-called Respiratory Distress Syndrome (RDS) of the newborn. This natural substance, generated from week 25 of pregnancy approximately, plays an important role in increasing the pulmonary compliance and preventing the collapse of the lung at the end of the expiration.

The surfactant replacement therapy employed currently consists of the instillation of exogenous surfactant and the application of mechanical ventilation. Even though this procedure provides good results, it is an invasive technique that may lead to deep lung and cerebral injuries, even with short terms of mechanical ventilation [1,2]. For that reason, over the last few years, research has been focused on the development of less invasive surfactant administration (LISA) techniques, by which natural surfactant can be delivered without the need of tracheal intubation [3]. Despite the recent advances in the development of new synthetic surfactants, as the lucinactant, the guidelines still recommend the application of animal-derived ones [4]. A survival advantage was noted when using a porcine lung surfactant dosage of 2.5 mL/kg with respect to 1.25 mL/kg of bovine lung surfactant, although it is uncertain if this was a consequence of the dose difference or of the composition of each type of surfactant [5].

Within the different LISA techniques, aerosolization emerges as a promising choice to alleviate the effects of the RDS in preterm babies. However, many issues have still to be overcome. Four clinical trials [6,7,8,9] showed the safety and feasibility of this technique, but only the work of Jorch et al. [7] provided an improvement of respiratory parameters and the avoidance of mechanical ventilation and endotracheal intubation. Pillow and Minocchieri [10] are currently working on a randomized controlled trial where the infant receive aerosolized surfactant in the first hour of life. Their goal is to analyze the need for intubation in the first 72 h of life and the duration of mechanical ventilation in this period. The main issue to overcome with the aerosolized surfactants is the small proportion reaching the lungs. Kohler et al. [11] made a comparison with jet and ultrasonic nebulizers obtaining a lung deposition not higher than 1% of the initial dose.

Perfluorocarbons (PFCs) have shown great potential to treat pulmonary failure in neonatal, pediatric, and adult animal models, reaching clinical trials. PFCs have been investigated in the setting of acute pulmonary failure because of their low surface tension (surfactant action), a relatively high solubility of respiratory gases, and a high density. Kandler et al. [12] studied the effect of aerosolized PFC in a surfactant-depleted piglet model, showing an improvement of oxygenation and pulmonary gas exchange. The subsequent work of von der Hart et al. [13] supported these results and confirmed the suitability of different PFCs for aerosol treatment. Burkhardt et al. [14] confirmed that the administration of an emulsion of surfactant and PFC contributes to obtain a more homogenous distribution and an improvement in oxygenation. Murgia et al. [15,16] studied the suitability of intratracheal inhalation catheters to produce the aerosolization of surfactant or PFCs during different mechanical ventilation strategies. Goikoetxea et al. [17] analyzed the feasibility of delivering aerosolized surfactant and PFCs beyond the third generation of branching in a preterm infant airway model by means of an inhalation catheter. Recently, Syedain et al. [18] developed a novel aerosol generator to deliver surfactant in preterm infants with a low air flow, ideal to prevent potential lung injuries.

The definition of numerical models by means of Computational Fluid Dynamics (CFD) tools provides a helpful methodology to analyze several health issues. Oldham et al. [19] studied the influence of parameters such as the breathing rate and the particle size on particle deposition in the respiratory tract. Kleinstreuer et al. [20] made a review of inhaled toxic aerosols in cigarette smoke, marking the importance of CFD simulation models as a promising research tool to study all physical mechanisms involved in the deposition and transport of toxins in models of the respiratory system. Koombua et al. [21] explored the influence of airway wall elasticity analyzing two cases, rigid and flexible wall, respectively. It was observed that the pressure within the airways was affected but not the air flow velocity or the wall shear stress. Feng and Kleinstreuer [22] studied the deposition, interaction, and transport of particles in triple bifurcations, by means of the dense discrete phase model and the discrete element method. Recently, Elcner et al. [23] developed a CFD model validated with experimental results of the inspiratory airflow in a model from the throat to the fourth generation of the respiratory tract.

Nevertheless, most of the CFD literature is based on the geometries of the adult airways. The smaller size of the preterm infants’ airways and the difference of breathing conditions affect parameters such as the air flow velocity and particle transport and, therefore, do not make it possible to extrapolate the results obtained from adult airways. In the treatment of respiratory diseases, Longest et al. [24] created a CFD numerical model to evaluate the deposition patterns in the airways of a four-year-old child under healthy and constricted conditions. Liu et al. [25] studied, with a CFD model, the airflow patterns and the deposition of particles in the first three generations of a pediatric upper respiratory tract. Their results showed higher velocity fields, deposition rates, and impaction numbers in comparison with those observed in adults. De Jongh et al. [26] obtained, by means of computed tomography, the geometry of the upper airways of a nine-month-old child to calculate the deposition of microparticles and compared them with experimental results. The aerosolization of surfactant was also analyzed with a CFD model by Goikoetxea et al. [27] using an inhalation catheter with minimal manipulation of the airways.

In this article, an experimental and numerical model of the aerosol produced with an inhalation catheter has been developed. Two perfluorocarbons compounds are analyzed, PFD and FC75, at driving pressures of 4–5 bar, and the numerical results are compared with experimental data. 

## 2. Experimental Setup

**Figure 1:**
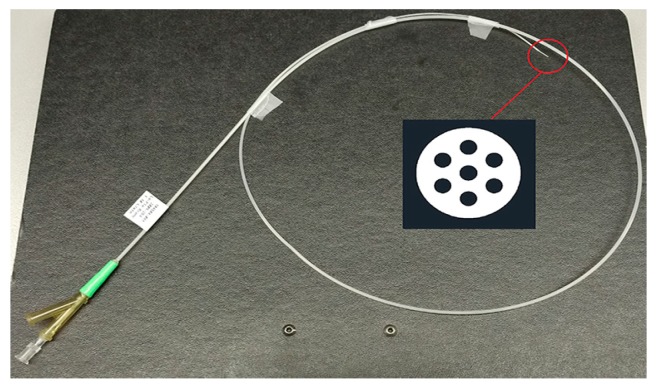
Inhalation catheter (IC 1.1) with a cross section detail of the distal end.

The experimental model to produce the aerosol was carried out with an inhalation catheter (IC), as shown in Figure 1. It consists of a central lumen, where the liquid to be aerosolized is delivered, and six outer lumens where compressed air is dispensed. The diameter of these lumens becomes smaller as it approximates to the catheter tip, resulting in an increase of the air velocity. This high air velocity, along with the closeness of the lumens, leads to the aerosolization of the fluid. In the present study, two different perfluorocarbons (PFC), perfluorodecalin (PFD; density = 1.95 g/mL) and FC75 (density = 1.78 g/mL) were used to produce aerosols with driving pressures of 4–5 bar.

**Figure 2:**
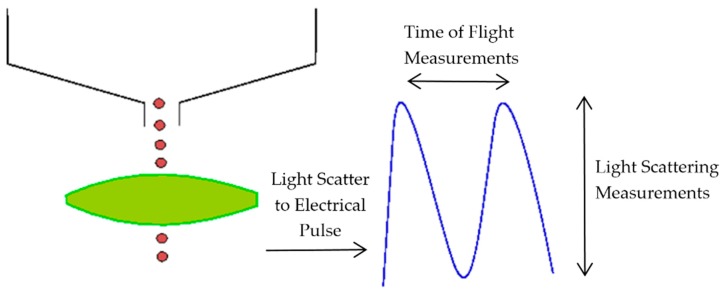
Double-crested signal generated by the Aerodynamic Particle Sizer (APS) laser beams.

The aerosol parameters were measured by an Aerodynamic Particle Sizer (APS) spectrometer, which provides high-resolution measurements of aerosol particles from 0.5 to 20 µm. It uses a patented double-crested optical system for unmatched sizing accuracy and, by means of two laser beams, it generates a signal when a particle passes through them, as illustrated in Figure 2. The acceleration of particles, that will be smaller for bigger particles because of their larger inertia, is obtained from the time between the peaks of the signal, known as time-of-flight. Then, with the sphere calibration stored in the spectrometer memory, the APS converts each time-of-flight measurement to its corresponding aerodynamic particle diameter. For a particle, the aerodynamic diameter is described as the diameter of a spherical particle with a density of a water droplet (1000 kg/m^3^) that has the same settling velocity as the particle. 

**Figure 3:**
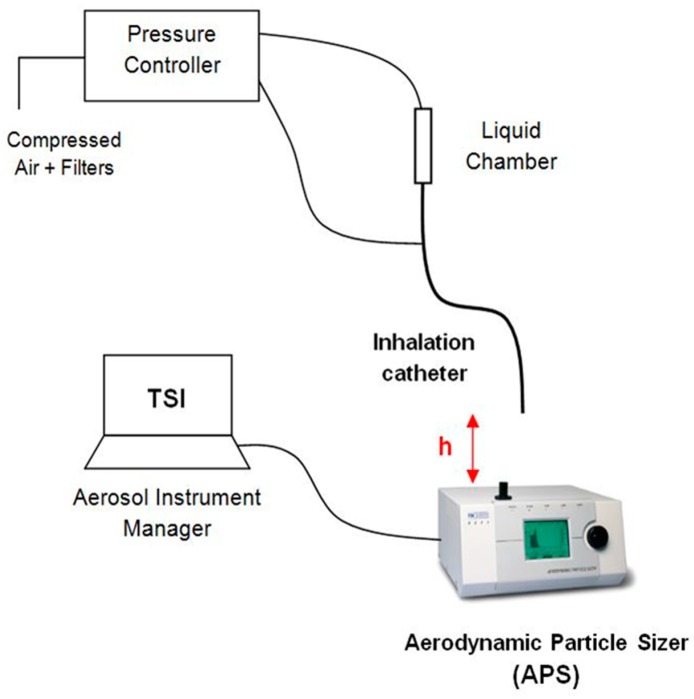
Experimental setup for particle size characterization.

Figure 3 illustrates the experimental setup used to measure the aerosol properties on the basis of the study of Aramendia et al. [28]. A pressure regulator was used to provide compressed air to both the liquid chamber and the connection directed to the outer lumens of the IC. The distance between the catheter tip of the IC and the inlet nozzle of the APS was checked in order to obtain the average 1000 particles/cm^3^ particle concentration recommended by the manufacturer. The APS measures and classifies the particles in four events according to their aerodynamic diameter. The first one groups the particles with a diameter smaller than 0.5 µm, the second one classifies the particles that are in the spectrometer measuring range from 0.5 to 20 µm, the third event considers those particles that cross the laser beams at the same time, and the last event catches particles bigger than 20 µm. Thus, in order to get accurate measurements, it is important to get the vast majority of the particles classified in the second group. All the information and parameters captured by the APS were recorded and stored by the software associated with the hardware.

## 3. Experimental Results

The compounds PFD and FC75 were tested at driving pressures of 4–5 bar, while varying the distance between the APS nozzle and the catheter tip. The results are summarized in Table 1, Table 2, Table 3 and Table 4. A sample time of 10 s was set, recording five samples for each pressure and distance. The optimal distance was achieved taking into account three criteria. Firstly, that most of the particles measured were classified in the first and second event of the APS, ensuring that the particle coincidence or recirculating particles did not affect the accuracy of the measurements; secondly, that the particle concentration was within the recommended range according to the APS manufacturer guideline; finally, that a minimum of five samples were obtained in this previous conditions for each pressure and compound. It was found that the optimal distance was 60 mm and 52 mm for PFD and FC75, respectively. Initially, for FC75, measurements were taken at higher distances, however, a very low particle concentration was observed that could be explained by the high volatility of the compound. Additionally, measurements at 30 mm and 46 mm were taken for the PFD compound to compare the results at different distances. 

Three parameters were analyzed to study the aerosol behavior. Firstly, we analyzed the geometric standard deviation (GSD), which is an indicator of the spread of an aerodynamic particle size distribution. Values below 1.25 denote an aerosol formed by particles of approximately the same size, whereas values above 1.25 indicate an aerosol made up of particles with different sizes. Another important parameter is the mass median aerodynamic diameter (MMAD), which measures the aerodynamic diameter at which 50% of the aerosol mass will be present in particles below this value. Finally, the geometric particle diameter (D_g_) was analyzed. This value can be obtained with the aerodynamic diameter (D_a_) measured with the APS by the expression given by Equation (1):

(1) $$D_{g}=D_{a}\sqrt{\frac{\rho_{0}}{\rho }}$$

where *ρ* is the density of the PFC, *ρ* = 1.95 g/cm^3^ for PFD and *ρ* = 1.78 g/cm^3^ for FC75, respectively, and *ρ*_0_ is the unit density, 1 g/cm^3^.

These experimental results were used subsequently to validate the numerical model. Furthermore, measurements at the nozzle level (h = 0) were taken to define the initial conditions of the injectors that will represent the population of the particles in the numerical model. The average particle concentration measured at this point was higher than the recommended. However, the vast majority of them were grouped in the second event, which measures the particles within the range of the APS from 0.5 to 20 µm.

The PFD and FC75 aerosolization rate (AR) was measured by means of the Equation (2).

(2) $$\mathrm{AR}=\frac{{\left(m_{cam}\right)}_{t}-{\left(m_{cam}\right)}_{0}}{t_{pulse}\times \rho }$$

where 0 correspond to the instant before and *t* to the instant after the aerosolization pulse, *m_cam_* is the mass of PFC within the liquid chamber, *ρ* is the density of the PFC, and *t_pulse_* is the time of the aerosolization pulse.

An AR of 0.272 mL/min and 1.066 mL/min was obtained with a pressure of 4 bar for PFD and FC75, respectively. Similarly, the AR corresponds to 0.303 mL/min and 1.313 mL/min for a pressure of 5 bar. As it was expected, an increase of the pressure resulted in higher AR values.

## 4. Numerical Model

Computational Fluid Dynamics (CFD) tools have emerged as a viable approach for either industrial and research applications as a resultof the constant improvement of digital computers and computational resources. With the introduction of new solvers and theoretical physic models, CFD techniques allow to study alternative designs under a wide range of parameters and conditions. In this article, the commercial CFD software STAR-CCM+ v.11.06 (Siemens^®^, London, UK) [29] was employed to define and solve the numerical model of the aerosol produced by the inhalation catheter.

### 4.1. Computational Domain

**Figure 4:**
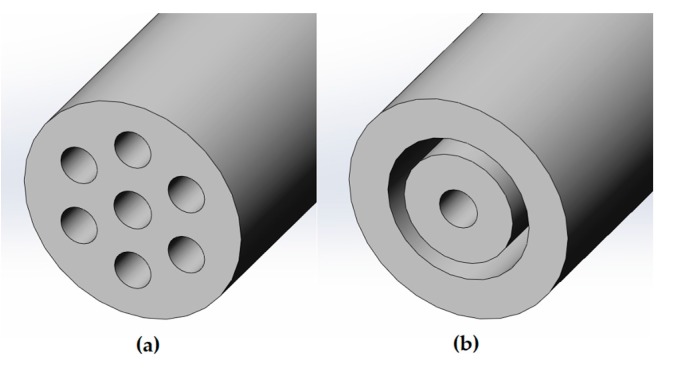
Cross section of the IC 1.1 tip (**a**) and the assumption defined for the generation of the axisymmetrical computational domain (**b**).

The Lagrangian multiphase simulations involve a high computational cost due to the solution of the trajectory of each of the discrete particles of the aerosol. For that reason, it was assumed an axisymmetrical computational domain with an outer ring instead of the six original lumens (See Figure 4). The width of this ring was computed in order to be of the same area of the six lumens and, therefore, to supply the same air mass flow.

**Figure 5:**
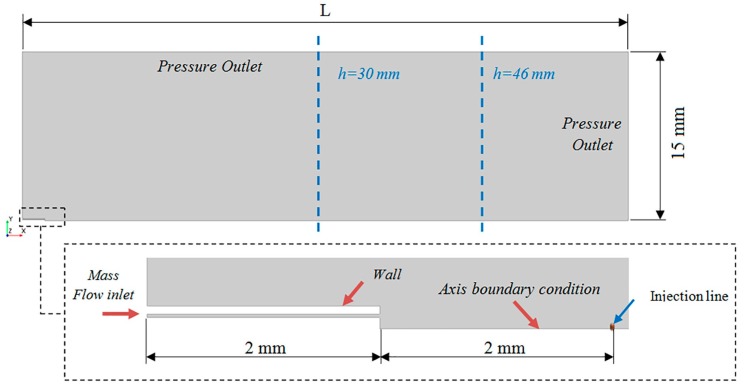
Computational domain and boundary conditions.

The computational domain was created with the last 2 mm of the IC and the region downstream of the catheter tip, as shown in Figure 5. The optimal distance used in the experimental setup to obtain the average recommended particle concentration with the APS was used to define the outlet boundary condition, which was located at L = 60 mm and L = 52 mm for PFD and FC75, respectively. 

An air mass flow rate of 1.1344 × 10^−5^ kg/s, previously measured with a pressure of 4 bar in the work of Goikoetxea et al. [17], and of 1.14766 × 10^−5^ kg/s with a pressure of 5 bar were used to define the inlet boundary condition. In addition, an axisymmetrical boundary condition was fixed along the central axis, and an atmospheric outlet condition was set for the downstream boundaries. For the representation of the discrete phase particles, ten particle injections were created and uniformly distributed through an injection line, whose length was equal to the radius of the central lumen of the IC. The ten injections were defined by interpolating the data from the 52 intervals of the APS sample chosen. They were set at a distance of 2 mm from the catheter tip as an approximated value between the APS nozzle, where the catheter tip was placed to take the measurements, and the APS laser beams which measure the distribution of the particles. The particle mass and size distribution values of these initial injections, obtained from experimental measurements, are defined in Table 5 for each PFC compound. 

### 4.2. Discretization 

**Figure 6:**
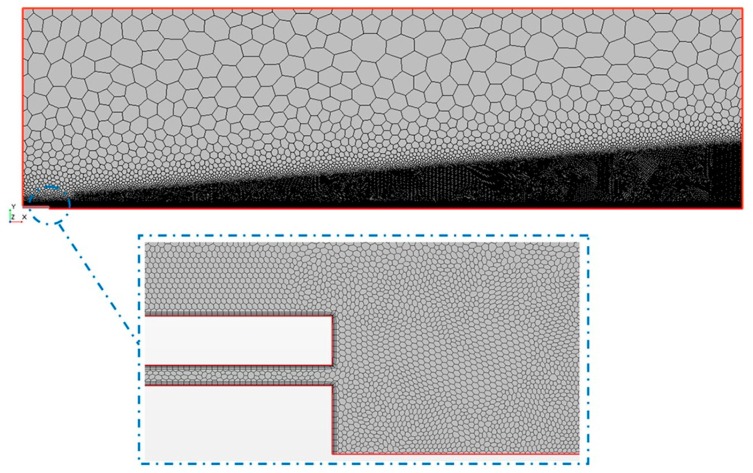
Discretised representation of the computational domain.

The mesh generation represents one of the most important steps during the pre-process part before running the numerical solution. The CFD code requires the division of the computational domain in smaller subdomains in order to solve the flow physics. In this study, polygonal cells were used with a mesh refinement in the region close to the catheter tip and all along the central axis, as shown in Figure 6.

To ensure that the numerical solution is independent of the mesh size, a mesh dependency study was carried out following the procedure of the Richardson’s extrapolation [30]. Three different meshes were created (coarse, medium, and fine) with mesh sizes h_3_, h_2_, and h_1_, respectively. The mesh refinement ratio is given by Equation (3):

(3) $$\mathrm{Mesh}\;\mathrm{refinement}\;\mathrm{ratio}=r=\frac{h_{2}}{h_{1}}$$

The axial velocity in a point at the outlet was the parameter chosen to control the study. Table 6, Table 7 and Table 8 show the results obtained for the FC75 compound with a driving pressure of 5 bar.

The extrapolated axial velocity value was calculated by Equation (4), where p is defined as the order of accuracy given by Equation (5):

(4) $${\left(v_{\mathrm{axial}}\right)}_{h=0}={\left(v_{\mathrm{axial}}\right)}_{1}+\frac{{\left(v_{\mathrm{axial}}\right)}_{1}-{\left(v_{\mathrm{axial}}\right)}_{2}}{r^{p}-1}$$

(5) $$p=\frac{\ln\left(\frac{{\left(v_{\mathrm{axial}}\right)}_{3}-{\left(v_{\mathrm{axial}}\right)}_{2}}{{\left(v_{\mathrm{axial}}\right)}_{2}-{\left(v_{\mathrm{axial}}\right)}_{1}}\right)}{\ln\;2}$$

The discretization error of the computed solution was calculated using the grid convergence index (GCI) with the three levels of mesh previously defined (6) and (7). A small value of GCI indicates that we are within the asymptotic range of convergence [31].

(6) $$\begin{array}{c} {\mathrm{GCI}}_{12}=F_{s}\frac{\left|{\left(v_{\mathrm{axial}}\right)}_{1}-\frac{{\left(v_{\mathrm{axial}}\right)}_{2}}{{\left(v_{\mathrm{axial}}\right)}_{1}}\right|}{r^{p}-1}\times 100\\ {\mathrm{GCI}}_{23}=F_{s}\frac{\left|{\left(v_{\mathrm{axial}}\right)}_{2}-\frac{{\left(v_{\mathrm{axial}}\right)}_{3}}{{\left(v_{\mathrm{axial}}\right)}_{2}}\right|}{r^{p}-1}\times 100 \end{array}$$

(7) $$\frac{{\mathrm{GCI}}_{23}}{r^{p}\cdot {\mathrm{GCI}}_{12}}\approx 1$$

The mesh level M1, with 404.620 cells, was chosen to create the numerical model taking into consideration the error obtained for each mesh level, and that the GCI_23_/rp·GCI12 relation was close to 1, see Table 7 and Table 8.

The definition of a temporal discretization was also necessary because of the unsteady condition of the aerosol. The time-step was set to 1 × 10^−4^ s, and 300 inner iterations per time-step were defined in order to obtain the convergence of the numerical solution. 

**Figure 7:**
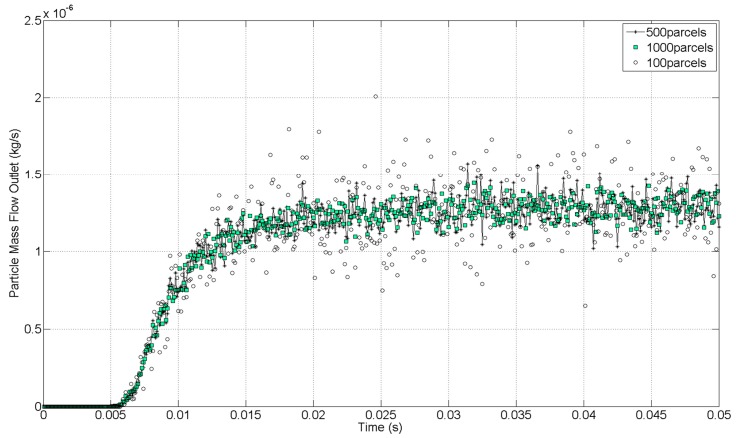
Particle mass imbalance at the outlet with different numbers of computational parcels.

From the computational standpoint, solving the trajectories of hundreds of thousands of physical particles is extremely expensive. For this reason, the CFD code uses parcels to reduce the computational cost. Each parcel represents a group of droplets that have the same properties. Therefore, the total population of particles was represented by a smaller number of computational parcels. Just as with cells, the number of parcels is not arbitrary; it must be large enough so that the properties of the full population of dispersed phases are represented. In that sense, a similar dependency study is necessary to control the discretization accuracy of the particle population. This is visible from a monitor plot of the particle mass flow at the outlet of the computational domain. The results for the PFD compound at 4 bar were monitored with three different number of parcels injected per time-step (100 parcels/Δt, 500 parcels/Δt, and 1000 parcels/Δt). A time-step of 1 × 10^−4^ s was defined for all cases. The initial mass flow inlet, defined as a boundary condition, corresponded to 1.28 × 10^−6^ kg/s. The condensation and evaporation of the discrete phase were not taken into account in this study, thus, according to the conservation of mass, the particle mass flow at the outlet has to match the particle mass flow defined in the inlet. An increase of the number of parcels injected improves the accuracy of the solution, as can be seen in Figure 7. A balance between the accuracy of the solution and the computational time was needed. A considerable difference was observed between the case with 100 and 500 parcels per time-step. Because of the high difference in computational time between the cases of 500 and 1000 per time-step and the results obtained, the case with 500 parcels per time-step was considered as the best relation between accuracy and computational cost to discretize the particle population.

### 4.3. Physics and Numerical Methods

The governing equations for the continuous phase were expressed in Eulerian form, whereas the Lagrangian description allowed to solve the dispersed phase as it crossed the computational domain. In a Lagrangian framework, particle-like elements known as parcels, represents the true number of particles of the dispersed phase and are introduced in the computational domain by means of injectors. This model is applied in the case of dilute sprays where the continuous phase transports a relatively small volume of droplets. For instance, in the CFD code used in this study, the volume fraction of the Lagrangian phase in each cell had to be smaller than 40%.

The motion of the dispersed droplets was solved by means of the Newton’s second law. The drag force, defined by Equation (8), calculates the force on a droplet due to its velocity relative to the continuous phase. The drag coefficient CD in the Relation (8) is a function of the small-scale flow features around the individual particles. These features are impractical to resolve spatially, and so the usual practice is to obtain the drag coefficient from correlations. In the current work, the Schiller–Naumann correlation, suitable for liquid droplets, was employed:

(8) $$F_{D}=\frac{1}{2}C_{D}\rho A_{p}v_{\mathrm{rel}}^{2}$$

where *ρ* is the density of the air flow, CD the particle drag coefficient, vrel the relative velocity, and Ap the projected area of the particle.

According to Crowe et al. [32], the fact that the droplets density is much larger than the surrounding fluid density results in the drag force being much larger than all the other fluid forces acting on the droplet. For that reason, only the drag force was included in the numerical simulation, while virtual mass and basset history terms, as well as pressure gradient forces were neglected (see also Zhang et al. [33]). The influence of the gravity force was considered insignificant in comparison with the high velocity gradients in the outlet of the catheter tip.

The high air velocity in the region close to the catheter tip contributes to the appearance of turbulent eddies and perturbations that can eventually lead to a random state of motion. In this article, the two-layer realizable k-epsilon model was used in order to solve the turbulent condition of the flow [34]. Regarding the discrete phase, a particle in a turbulent flow experiences a randomly varying velocity field to which it responds according to its inertia. Parcels that are introduced into a turbulent carrier flow each have their own random path due to the interaction with the fluctuating turbulent velocity field. This behavior was modeled by a stochastic approach based on the work done by Gosman and Ioannides [35] that includes the effect of instantaneous velocity fluctuations on the particle. The characteristic size of the randomly sampled eddy is the dissipation length scale (l_ε_). For fully developed duct flows, it is given by Equation (9):

(9) $$l_{\varepsilon }=0.07\times L$$

where L is the width of the outer ring. With the dissipation length scale already known, the turbulent dissipation rate can be determined from the Relation (10)

(10) $$\varepsilon =C_{\mu }^{1/2}\frac{k^{3/2}}{l_{\varepsilon }}$$

where k is the turbulent kinetic energy, ε is the turbulent dissipation rate, and C_µ_ is an empirical constant of the turbulent model with a value of 0.09.

Furthermore, a two-way coupling model was used to calculate the interactions between the airflow and the PFC droplets. The atomization results from the interaction of the liquid jet with high-velocity compressed air. Because of the high velocity gradients in the outlet of the catheter tip, the primary atomization was assumed to occur before the particle injectors. The particle injectors were placed 2 mm away from the catheter tip according to the estimation of the distance between the APS nozzle and the laser beams that measures the aerosol properties. It must be noted that the APS has the potential to measure the properties of droplets between 0.5–20 µm, associated with the secondary atomization, and not the fragmentation and formation of ligaments related to the primary atomization. The coalescence of particles was not considered in this study, and the secondary breakup was defined by the Taylor Analogy Breakup (TAB) model [36], which is based on Taylor’s analogy. This model represents a distorting droplet as a damped spring–mass system, considering only the fundamental mode of oscillation of the droplet. The displacement and velocity of the mass in the spring–mass system correspond to representative distortion and rate of distortion quantities of the droplet. When the droplet oscillations reach a critical value, a breakup replaces the parent particles with child particles whose diameter is chosen from a Rosin–Rammler distribution. Despite being based on a single mode of oscillation in the vibrational regime, the TAB model reproduces the same characteristic time scales in low and high Weber number limits as the Reitz–Diwakar model (20 < *We* < 100), with the Weber number (*We*) given by (11):

(11) $$We=\frac{\rho_{g}u_{rel}^{2}D_{p}}{\sigma }$$

where $\rho_{g}$ is the density of the continuous phase, $u_{rel}$ is the relative velocity between the liquid droplet and the gas phase, *σ* is the surface tension, and $D_{p}$ is the particle diameter.

Typically, the TAB model is used at low Weber numbers. Outside its range of validity, the model tends to underpredict droplet sizes. In the present study, however, the Weber number was below 100 in all the cases studied.

## 5. Numerical Results and Experimental Validation

Firstly, the Navier–Stokes and continuity equations were solved for the continuous phase in steady state. The results of the converged solution were saved and subsequently used as the initial condition for the transient simulation of both phases, i.e., the airflow and the parcels representing the discrete phase.

**Figure 8:**
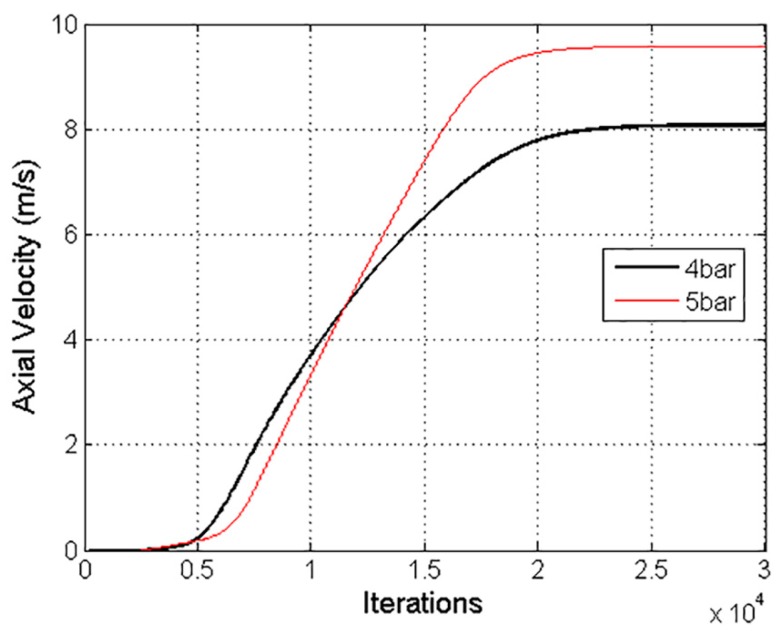
Axial air velocity values with pressures of 4 bar and 5 bar, considered to confirm the convergence of the numerical solution.

The solution was considered converged with a three-order-of-magnitude drop in the numerical residuals. Furthermore, an axial velocity variation of 8.7 × 10^−3^% in the last 1000 iterations confirmed the stability of the solution, as shown in Figure 8. A Courant number of 50 was defined, and the under-relaxation factor of the two-way coupling model was decreased to 0.1.

**Figure 9:**
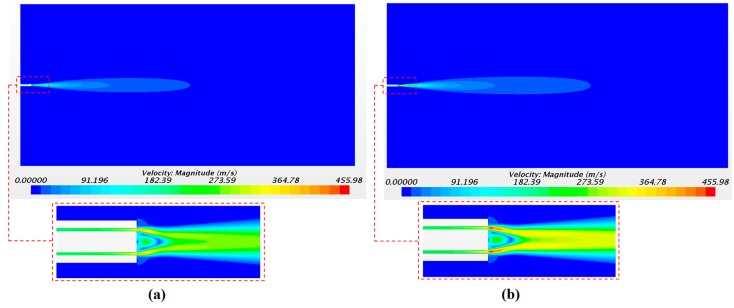
Continuous-phase velocity magnitude with a driving pressure of 4 bar (**a**) and 5 bar (**b**).

The solution of the continuous phase is illustrated in Figure 9 with the airflow velocity fields. Figure 9a,b corresponds to the velocity field with a driving pressure of 4 bar and of 5 bar, respectively. As expected, the largest values of the axial velocity were observed close to the catheter tip with 391 m/s and 455 m/s for pressures of 4 and 5 bar, respectively. The velocity in the injectors’ position was 247 m/s and 293 m/s for 4 and 5 bar of pressure, respectively. These values were checked in order to define the initial velocity condition of the droplets in the injector points. The high air velocity values obtained confirm the importance of considering the airflow as a compressible flow in this application.

**Figure 10:**
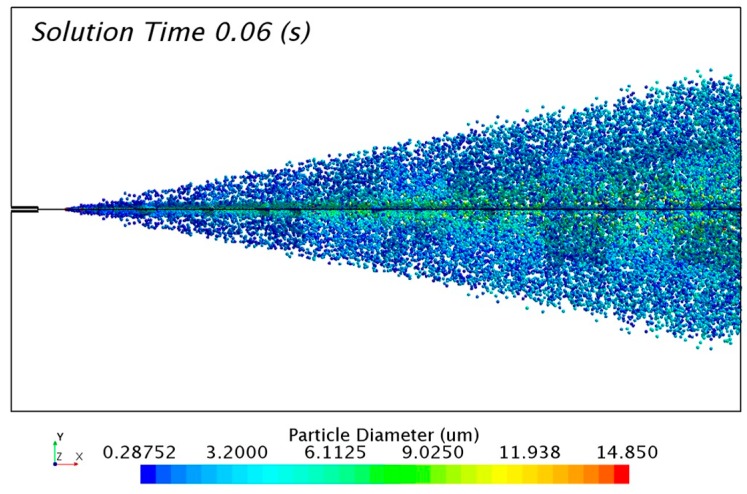
Particle size distribution of the FC75 discrete particles with a driving pressure of 4 bar.

The solution of the discrete phase is presented in Figure 10 with the formation of the aerosol cone. The solution was taken 0.06 s after the release of particles from the injectors. The transitory condition of the aerosol flow changes the distribution of particles with time. For that reason, it was necessary to track a representative sample of 0.01 s in order to study the numerical results.

**Figure 11:**
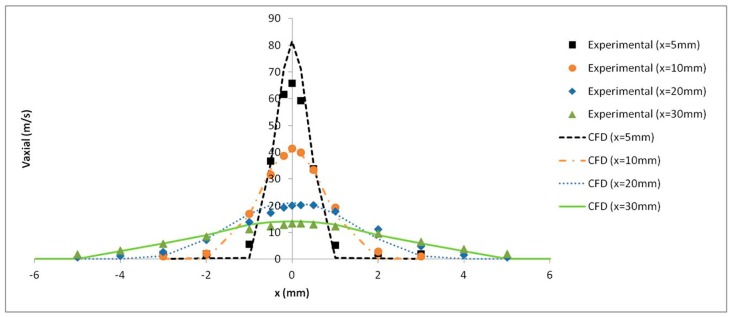
Experimental and numerical axial air velocity profiles at different distances from the catheter tip.

The axial velocity results with a pressure of 4 bar were monitored at several distances from the catheter tip and validated with the experimental results obtained by Goikoetxea et al. [37]. The turbulence model along with the mesh refinement generated close to the catheter tip led to an accurate prediction of the airflow velocity values, as shown in Figure 11.

**Figure 12:**
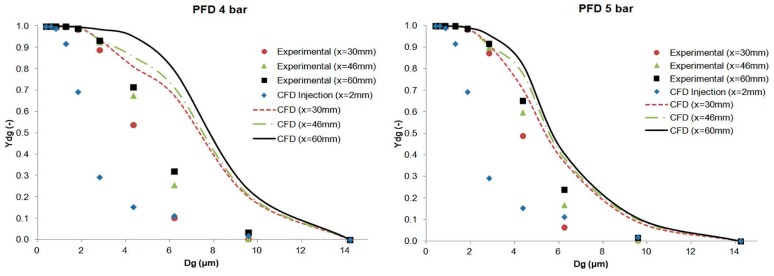
Experimental and numerical cumulative mass distribution based on droplet size for the PFD compound.

**Figure 13:**
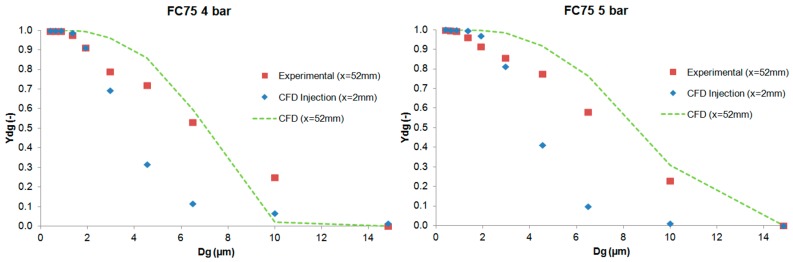
Experimental and numerical cumulative mass distribution based on droplet size for the FC75 compound.

The results of the cumulative mass distribution as a function of the droplet size (*Y_dg_*) are presented in Figure 12 and Figure 13 for PFD and FC75 compounds, respectively, and were compared with the experimental data obtained by means of the APS. Some differences were found between the numerical and the experimental results. The collision and coalescence of the particles may be the cause of those deviations. In the numerical model, which considered the breakup of particles with the TAB breakup model but not the coalescence, a higher cumulative mass fraction was obtained for particles above 3 µm for the PFD compound at both pressures and for each distance. For the FC75 compound, with a pressure of 4 bar, the cumulative mass fraction was higher for particles below 5 µm and considerable lower for particles above 10 µm, whereas with a driving pressure of 5 bar there was a different pattern in the experimental results, and the cumulative mass fraction for particles above 10 µm was considerable higher.

## 6. Conclusions

This article presents the generation of a PFC aerosol by means of an inhalation catheter and its main parameters measured with an Aerodynamic Particle Sizer (APS) and CFD tools. Different cases were studied for PFD and FC75 varying the driving pressure of the compressed air between 4 and 5 bar. The results were measured at the optimal distances between the APS nozzle and the catheter tip and at some additional distances in the case of PFD. The GSD values varied from 1.61 to 2.04, leading to the formation of heterodisperse aerosols. The aerodynamic diameter (D_a_) provided values that were within the recommended range values (1–5 µm) and, although the MMAD results were between 5–10 µm, we must take into account that the goal of this study was the generation of an aerosol beyond the nasopharyngeal area, avoiding in that way the deposition of these bigger particles.

Subsequently, the numerical model was introduced in order to simulate the generation of the PFD and FC75 aerosols with CFD techniques. These numerical simulations were validated with the axial velocity results obtained experimentally with a hot-wire anemometer, confirming their accuracy at different downstream positions. The differences in the cumulative mass distribution of the aerosol particles between the numerical and experimental procedure suggest that the collision and coalescence of the PFC droplets play an important role during the formation of the aerosol. Similar behavior could be seen for PFD at both driving pressures, whereas for FC75, the cumulative mass fraction for particles above 10 µm was considerable higher with a driving pressure of 5 bar.

The numerical study presented in this work could be a preliminary tool to facilitate parametric studies of optimal inhalators in preterm babies with RDS.

## Figures and Tables

**Table 1:** Perfluorodecalin (PFD) experimental results at h = 30 mm.

PFD 4 bar (h = 30 mm)				PFD 5 bar (h = 30 mm)			
Sample	Da (µm)	MMAD (µm)	GSD	Sample	Da (µm)	MMAD (µm)	GSD
1	3.96	7.07	1.99	6	3.97	6.27	1.80
2	4.13	6.61	1.76	7	3.99	6.33	1.82
3	4.09	6.46	1.73	8	3.98	6.32	1.82
4	4.12	6.55	1.74	9	3.93	6.21	1.82
5	4.09	6.50	1.74	10	3.96	6.36	1.84
MEAN	4.08	6.64	1.79	MEAN	3.97	6.30	1.82
SD	0.07	0.25	0.11	SD	0.02	0.06	0.01

Da denotes the aerodynamic diameter, MMAD denotes the mass median aerodynamic diameter, GSD denotes the geometric standard deviation and SD denotes the standard deviation.

**Table 2:** PFD experimental results at h = 46 mm.

PFD 4 bar (h = 46 mm)				PFD 5 bar (h = 46 mm)			
Sample	Da (µm)	MMAD (µm)	GSD	Sample	Da (µm)	MMAD (µm)	GSD
11	4.24	7.37	2.03	16	4.18	6.84	1.85
12	4.31	7.46	2.06	17	4.32	7.14	1.94
13	4.24	7.44	2.03	18	4.30	7.24	2.01
14	4.26	7.45	2.04	19	4.22	7.30	2.07
15	4.31	7.48	2.02	20	4.20	7.37	2.09
MEAN	4.27	7.44	2.04	21	4.28	7.44	2.11
SD	0.04	0.04	0.02	22	4.10	6.83	1.85
				23	4.13	6.91	1.86
				MEAN	4.22	7.13	1.97
				SD	0.08	0.24	0.11

Da denotes the aerodynamic diameter, MMAD denotes the mass median aerodynamic diameter, GSD denotes the geometric standard deviation and SD denotes the standard deviation.

**Table 3:** PFD experimental results at h = 60 mm.

PFD 4 bar (h = 60 mm)				PFD 5 bar (h = 60 mm)			
Sample	Da (µm)	MMAD (µm)	GSD	Sample	Da (µm)	MMAD (µm)	GSD
24	4.30	7.24	1.95	29	4.18	7.20	1.99
25	4.36	7.22	1.89	30	4.22	7.12	1.95
26	4.30	7.19	1.93	31	4.20	7.17	1.95
27	4.33	7.21	1.91	32	4.16	7.10	1.93
28	4.35	7.20	1.91	33	4.16	7.09	1.94
MEAN	4.33	7.21	1.92	MEAN	4.18	7.14	1.95
SD	0.03	0.02	0.02	SD	0.03	0.05	0.02

Da denotes the aerodynamic diameter, MMAD denotes the mass median aerodynamic diameter, GSD denotes the geometric standard deviation and SD denotes the standard deviation.

**Table 4:** FC75 experimental results at h = 52 mm.

FC75 4 bar (h = 52 mm)				FC75 5 bar (h = 52 mm)			
Sample	Da (µm)	MMAD (µm)	GSD	Sample	Da (µm)	MMAD (µm)	GSD
34	2.47	10.50	1.70	39	2.28	5.06	1.62
35	2.45	10.10	1.69	40	2.32	4.89	1.62
36	2.44	10.30	1.70	41	2.27	4.88	1.60
37	2.45	9.73	1.68	42	2.27	4.60	1.60
38	2.43	9.42	1.68	43	2.27	4.74	1.60
MEAN	2.45	10.01	1.69	MEAN	2.28	4.83	1.61
SD	0.01	0.44	0.01	SD	0.02	0.17	0.01

Da denotes the aerodynamic diameter, MMAD denotes the mass median aerodynamic diameter, GSD denotes the geometric standard deviation and SD denotes the standard deviation.

**Table 5:** Particle initial conditions for the PFD (left) and FC75 (right) compounds obtained from experimental measurements.

	PFD			FC75		
Injection	Dg (µm)	ṁ (kg/s)		Dg (µm)	ṁ (kg/s)	
		4 bar	5 bar		4 bar	5 bar
1	0.375	1.43 × 10^−9^	6.08 × 10^−12^	0.392	5.44 × 10^−9^	2.53 × 10^−11^
2	0.577	9.02 × 10^−10^	1.49 × 10^−11^	0.604	4.27 × 10^−9^	8.35 × 10^−11^
3	0.828	1.18 × 10^−8^	2.77 × 10^−10^	0.866	5.04 × 10^−8^	1.48 × 10^−9^
4	1.275	9.35 × 10^−8^	4.82 × 10^−9^	1.335	3.84 × 10^−7^	2.48 × 10^−8^
5	1.825	2.87 × 10^−7^	3.30 × 10^−8^	1.910	1.09 × 10^−6^	1.61 × 10^−7^
6	2.810	5.14 × 10^−7^	1.91 × 10^−7^	2.945	1.91 × 10^−6^	9.76 × 10^−7^
7	4.330	1.79 × 10^−7^	5.77 × 10^−7^	4.535	1.00 × 10^−6^	2.49 × 10^−6^
8	6.205	5.26 × 10^−8^	4.64 × 10^−7^	6.495	2.46 × 10^−7^	1.93 × 10^−6^
9	9.555	1.21 × 10^−7^	1.44 × 10^−7^	10.000	2.57 × 10^−7^	5.52 × 10^−7^
10	14.19	2.31 × 10^−8^	1.72 × 10^−8^	14.850	7.81 × 10^−8^	6.37 × 10^−8^
Total mass flow rate		1.28 × 10^−6^	1.43 × 10^−6^		5.03 × 10^−6^	6.20 × 10^−6^

**Table 6:** Axial velocity values in the chosen point for each mesh level.

Mesh	Number of Cells	Vaxial (m/s)
M1	404.620	9.58
M2	196.107	9.41
M3	98.854	8.92

**Table 7:** Errors obtained for each mesh level.

	Vaxial (m/s)	Error (%)
**(V_axial_)_h = 0_ (m/s)**	9.67	
**M1**	9.58	0.93
**M2**	9.41	2.69
**M3**	8.92	7.76

**Table 8:** Grid Convergence Index (GCI) results.

	Domain
**GCI12 (%)**	1.21
**GCI23 (%)**	3.65
**GCI23/rpGCI12 (-)**	1.05
